# Update on cerebral small vessel disease: a dynamic whole-brain disease

**DOI:** 10.1136/svn-2016-000035

**Published:** 2016-10-25

**Authors:** Yulu Shi, Joanna M Wardlaw

**Affiliations:** 1 Centre for Clinical Brain Sciences, University of Edinburgh, Edinburgh, UK; 2 Department of Neurology, Zhongnan Hospital, Wuhan University, Wuhan, China

**Keywords:** Cerebral Small Vessel Disease, Lacunar Infarct, White Matter Hyperintensities, Blood Brain Barrier, Microvascular dysfunction

## Abstract

Cerebral small vessel disease (CSVD) is a very common neurological disease in older people. It causes stroke and dementia, mood disturbance and gait problems. Since it is difficult to visualise CSVD pathologies in vivo, the diagnosis of CSVD has relied on imaging findings including white matter hyperintensities, lacunar ischaemic stroke, lacunes, microbleeds, visible perivascular spaces and many haemorrhagic strokes. However, variations in the use of definition and terms of these features have probably caused confusion and difficulties in interpreting results of previous studies. A standardised use of terms should be encouraged in CSVD research. These CSVD features have long been regarded as different lesions, but emerging evidence has indicated that they might share some common intrinsic microvascular pathologies and therefore, owing to its diffuse nature, CSVD should be regarded as a ‘whole-brain disease’. Single antiplatelet (for acute lacunar ischaemic stroke) and management of traditional risk factors still remain the most important therapeutic and preventive approach, due to limited understanding of pathophysiology in CSVD. Increasing evidence suggests that new studies should consider drugs that target endothelium and blood–brain barrier to prevent and treat CSVD. Epidemiology of CSVD might differ in Asian compared with Western populations (where most results and guidelines about CSVD and stroke originate), but more community-based data and clear stratification of stroke types are required to address this.

## Introduction

The term ‘cerebral small vessel disease (CSVD)’ refers to a syndrome of clinical and imaging findings that are thought to result from pathologies in perforating cerebral arterioles, capillaries and venules. CSVD causes up to 45% of dementia, and accounts for about 20% of all stroke worldwide, 25% of ischaemic (or lacunar strokes), of whom about 20% are left disabled.^1^ Cognitive impairment, depression and gait problems are also frequently seen in patients with CSVD. The prevalence of lacunar stroke may be higher in patients in China where recent studies have suggested that lacunar infarction accounts for 38–46% of ischaemic stroke.^2^ ^3^

Generally, including in this review, CSVD is used to describe a series of imaging changes in the white matter and subcortical grey matter, including recent small subcortical infarct, lacunes, white matter hyperintensities (WMHs), prominent perivascular spaces (PVS), cerebral microbleeds (CMBs) and atrophy.^4^ Usually, recent small subcortical infarcts cause acute stroke symptoms, whereas other CSVD lesions are clinically more insidious and thus referred to as ‘silent’ lesions. However, the definitions and terms of these lesions have varied greatly among studies. For example, a recent review identified 159 different names for recent small subcortical infarcts, but these names like ‘lacunar infarct’ were also frequently used to describe lacunes^4^ ^5^ that were not necessarily related to symptoms and might have been due to haemorrhage. The substantial variation in the use of these terms has probably contributed to confusion and difficulties in interpreting previous research. Therefore, in 2013, an expert workgroup on CSVD proposed a list of standard terms to help avoid confusion and suggests that CSVD researchers should be encouraged to apply these terms in future studies.^4^ We will also use these terms in this review.

The different features of CSVD have long been regarded as different types of tissue changes. However, recent studies show that these features are correlated, are more likely to share common diffuse intrinsic small vessel pathologies, and are probably also more ‘dynamic’ than previously thought. Advances in imaging techniques have brought new insights into mechanisms of CSVD. In this review, we will summarise findings in recent clinical studies on CSVD, discuss CSVD mechanisms and explore emerging prevention and treatment options.

### Clinical lacunar stroke

**Figure 1:**
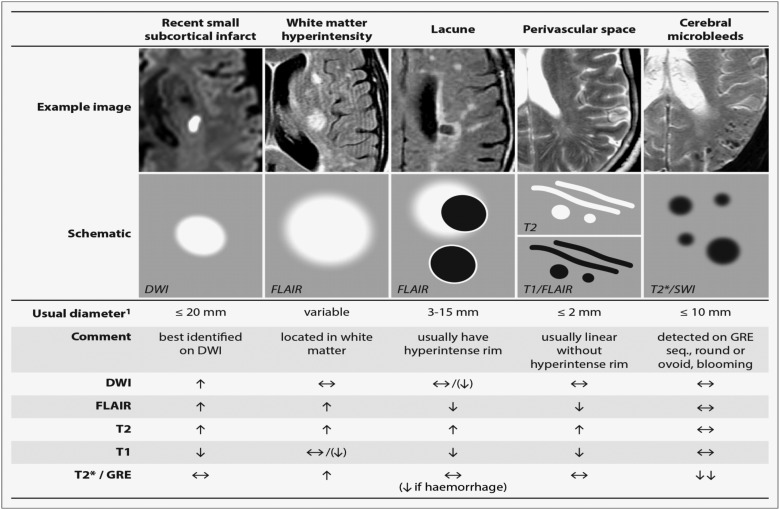
STRIVE, STandards for Reporting and Imaging of Small Vessel Disease: example findings (upper), schematic representation (middle) and a summary of imaging characteristics (lower) of MRI features for changes related to small vessel disease.^4^ DWI, diffusion-weighted imaging; FLAIR, fluid-attenuated inversion recovery; SWI, susceptibility-weighted imaging; GRE, gradient-recalled echo.

A lacunar clinical syndrome could be due to either ischaemia or a small haemorrhage.^6^ Many haemorrhagic strokes in older people are also due to CSVD pathology.^1^ In this review, we will focus mainly on ischaemic CSVD. Lacunar ischaemic stroke is defined as a stroke that is attributable to a recent small infarct <1.5 (or some say 2) cm diameter in the white matter, basal ganglia, pons or brainstem, and is consistent with a lacunar clinical syndrome.^7^ It is commonly attributed to an abnormality in a single small deep perforating (or lenticulostriate) artery. On MRI, an acute lacunar infarct is shown as hyperintense on diffusion-weighted imaging (DWI), hypointense on an apparent diffusion coefficient map, hyperintense on T2-weighted and fluid-attenuated inversion recovery (FLAIR), hypointense on T1 and hypoattenuated on CT (figure 1). It can be rounded, ovoid or tubular.^4^ Generally, the Oxfordshire Community Stroke Project (OCSP) classification, which uses only clinical features to diagnose the stroke subtype, can predict correctly the size and location of a recent brain infarct on imaging in 75–80% of patients with stroke.^8^ However, up to 20% of acute lacunar infarcts can present with cortical symptoms, and conversely cortical infarcts can present with lacunar syndromes.^9^ One explanation is that lacunar infarcts closer to the cortex are more likely to cause cortical symptoms.^9^ Therefore, in studies where stroke diagnosis relied mainly on the clinical presentations, this ‘mismatch’ may have added ‘noise’. Thus, in epidemiology, mechanistic studies or clinical trials, it is important to verify stroke lesions using sensitive imaging wherever possible.

However, even with sensitive imaging like DWI, about 30% of patients with clinically definite stroke did not show any recent ischaemic change on MRI;^10^ when followed up for a year, the DWI-negative patients had just as much recurrent stroke, dependency and cognitive impairment as the DWI-positive patients. Therefore, negative DWI/MRI cannot exclude stroke diagnosis. Rapid access to scanning after stroke onset can increase the chance of positive findings.^11^ It is also noteworthy that DWI-positive lesions can be clinically ‘silent’, for example, (1) as a second silent acute infarct in patients presenting with stroke due to another acute symptomatic infarct, or (2) in patients with acute haemorrhagic stroke, and (3) in patients with severe WMHs who did not have any overt stroke symptoms.^12^

**Figure 2:**
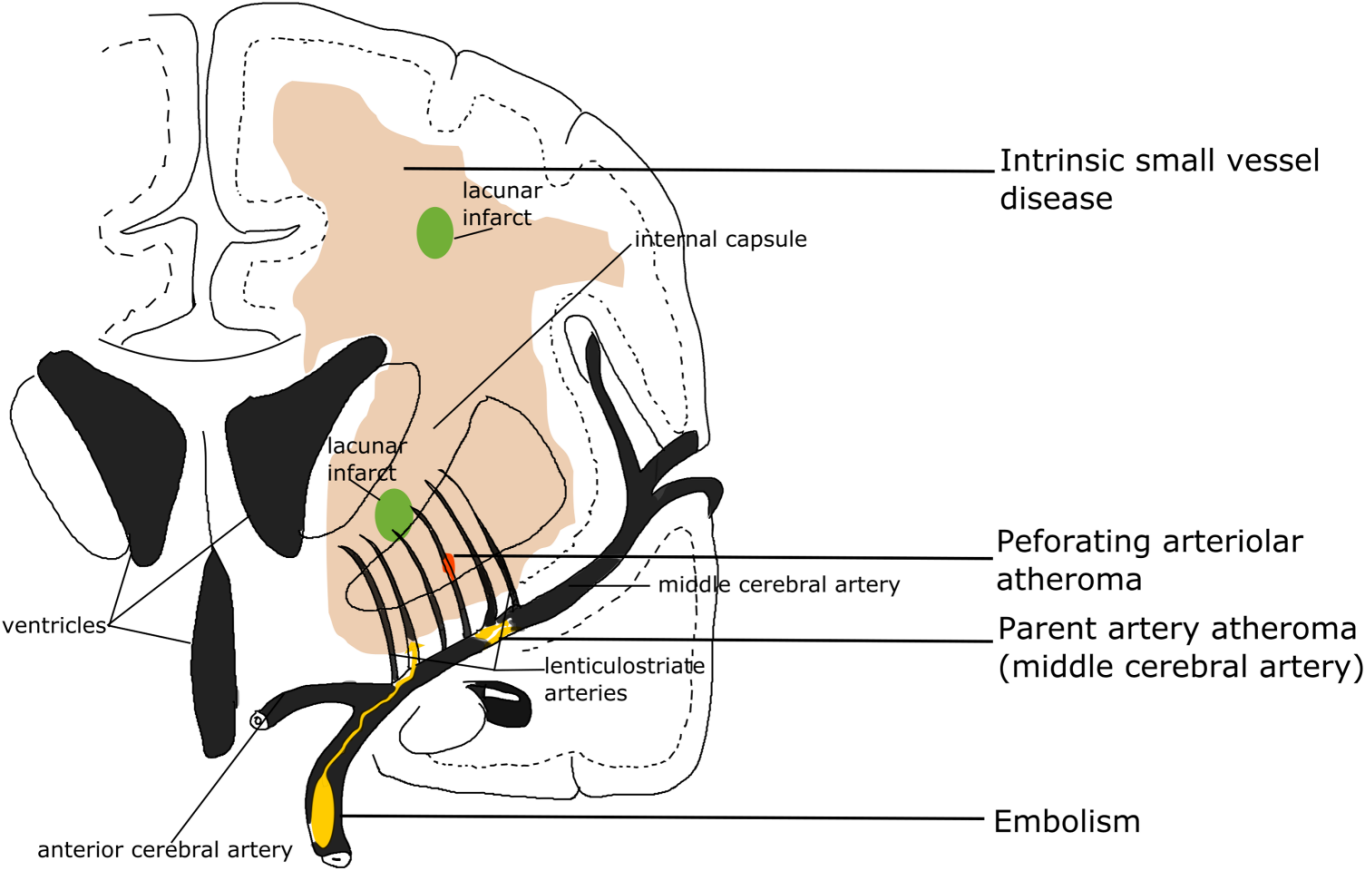
Four possible mechanisms that cause a lacunar infarct (from bottom to top): (A) an embolus from the big arteries or cardiac sources goes up to MCA and ends up entering and occluding lenticulostriate arteries, resulting in a lacunar lesion in basal ganglia; (B) if the atheroma in the parent artery (ie, MCA) is positioned at the opening of its penetrating branches, it could lead to an acute occlusion of one or several penetrating arteries, hence causing a lacunar infarct; (C) a lacunar infarct could also be due to atheroma in the perforating artery if an acute occlusion happens; (D) intrinsic small vessel disease may lead to diffused disrupted blood–brain barrier. If this happens at an arteriolar level, plasma fluid components would enter and deposit in the vessel wall, resulting in narrowing of the arteriolar lumen, vessel wall thickening and eventually a secondary luminal occlusion and traditional infarct. MCA, middle cerebral arteries.

In some clinical stroke classifications such as the Trial of Org 10172 in Acute Stroke Treatment (TOAST) or the ASCO (A: atherosclerosis; S: small-vessel disease; C: cardiac pathology; O: other causes), another term ‘small vessel/artery disease’ rather than ‘lacunar stroke’ is used to represent a stroke that is supposed to be due to a small artery occlusion. However, these classifications use risk factors to decide the stroke subtype, not just the clinical presentation, so as to distinguish ‘small vessel/artery disease’ from strokes caused by large artery atherosclerosis, cardiac emboli or other unknown reasons. However, a small embolus, or atheroma in the middle cerebral artery (MCA) or perforating arterioles can all block the perforating arteriole, and any of these can cause a lacunar ischaemic stroke (see figure 2). Therefore, it might be better to focus on the clinical presentation to assign the stroke syndrome and separately focus on the risk factors for patient management.

### Risk factors and causes of lacunar infarcts

Four possible main aetiologies for lacunar ischaemic stroke have been proposed (figure 2): atheroma of parent arteries (usually MCA) or perforating arterioles, embolism from the heart or carotid arteries, and intrinsic small vessel disease (lipohyalinosis or fibrinoid necrosis). Atheroma in MCA appears to cause <20% of lacunar ischaemic stroke. In the Warfarin Aspirin Symptomatic Intracranial Disease (WASID) trial, only 11% (38/347) of all patients with stroke were lacunar type,^13^ which is surprising if MCA stenosis is supposed to be a common cause of lacunar stroke. A recent study also did not find any association between lacunar stroke and MCA stenosis.^14^ A systematic review of Asian studies showed that parent artery atherosclerosis accounted for 20% of single lacunar infarcts in anterior circulation territory; however, these hospital-based studies were rather small (n=71–118) and some were even retrospective.^15^ Larger and tubular lacunar infarcts might be more likely to be caused by proximal artery diseases.^16^ However, the results of both our study and the Secondary Prevention of Small Subcortical Stokes Trial (SPS3) suggest that it is not possible to identify the cause of a particular recent lacunar ischaemic stroke based on its size, shape or location.^17^ ^18^

Evidence for embolism as a common cause for lacunar ischaemic stroke is limited. Presence of cardioembolic sources was found significantly less often in lacunar than in non-lacunar ischaemic stroke.^19^ ^20^ Few if any associations were found between ipsilateral carotid stenosis and lacunar ischaemic stroke or other features of CSVD.^21^ ^22^ In primate models, <6% of emboli injected into carotid arteries entered the lenticulostriate arteries, while the majority entered the cortical arteries.^23^ Lacunar ischaemic strokes in the basal ganglia were marginally more often associated with embolism than those in the centrum semiovale (11% vs 3%, respectively), but the overall rate of known embolic sources in symptomatic lacunar ischaemic stroke was very low (11%).^18^

Intrinsic small vessel pathologies remain the most common cause of lacunar ischaemic stroke, although the underlying mechanism is unclear. Fisher attributed the lipohyalinosis in small arteries to hypertension. However, the diagnosis and treatment of hypertension were less good when Fisher was working in the 1950s and 1960s and he may have seen some particularly severe cases of hypertension. Now, epidemiology data show that hypertension is equally common in non-lacunar as in lacunar ischaemic stroke;^19^ and many patients with lacunar stroke are normotensive. Similarly, other traditional risk factors like diabetes mellitus, hypercholesterolaemia and smoking were as frequent in lacunar stroke as in other ischaemic strokes.^24^ Risk factor profiles of lacunar stroke seemed different in China, but it might be too early to say so. The Beijing stroke registry (n=1184) showed a higher proportion of hypertension in lacunar (acute stroke symptoms+subcortical lesion <2 cm diameter on acute CT/MRI) than in non-lacunar stroke after adjusting for age and gender.^3^ Some other studies had similar findings, but the stroke diagnosis varied: in some studies, the differentiation between lacunar stroke and ‘large artery atherosclerosis’ stroke relied only on lesion size, and clinical classification included risk factors.^25^ ^26^ Additionally, most studies were hospital-based. Hence, population scale data on lacunar stroke are lacking. It is important to distinguish lacunar stroke from other subtypes because of the mechanism, hence prevention and treatment might differ. More data and careful separation of lacunar stroke from other subtypes are required in future studies.

### Clinically ‘Silent’ CSVD

#### White matter hyperintensities

WMH of presumed vascular origin are very common in older individuals and regarded as typical signs of CSVD. Symptoms of WMH develop insidiously, such as cognitive impairment, dementia and depression,^1^ but it almost triples the risk of stroke, doubles the risk of dementia and increases the risk of death.^27^

WMHs are usually symmetrically and bilaterally distributed in the white matter including the pons and brain stem, and also occur in deep grey matter. They appear hyperintense to the normal brain on T2 or FLAIR MRI (figure 1), and can be patchy or confluent depending on their stage in development and severity.

Owing to limited pathology studies, the underlying pathology of WMH remains imprecise. Demyelination, loss of oligodendrocytes and axonal damage were often reported. Diffusion tensor imaging studies provided indirect evidence for axonal damage and impaired white matter integrity in WMH.^28^ Indeed, recent evidence indicates that WMHs are rather heterogeneous, perhaps reflecting different disease stages. Reduced density of glia and vacuolation were observed in severe WMH suggesting end stage disease.^29^ Autopsy MRI studies also found oedema that suggests leakage of fluid from an impaired blood–brain barrier (BBB) in and around WMH.^30^ ^31^ Although these ‘white’ lesions have until now been treated as if they were all the same, different degrees of ‘whiteness’ might indicate different ‘stages of formation’—some very white WMHs are probably at the end stage of disease and irreversible once demyelination or axonal damage has happened; some perhaps less white lesions might be reversible if they are mainly interstitial fluid (ISF) imbalances before permanent tissue damage has occurred. These observations remain to be confirmed in larger studies. These microstructural changes happen in WMH, and are also present in normal appearing white matter (NAWM).^32^ ^33^ The white matter integrity in NAWM declines with increasing closeness to the edge of WMH^32^ and with more severe WMH.^34^

Multiple mechanisms underlying WMH such as incomplete infarct, chronic hypoperfusion and venous collagenous have been proposed, but evidence for each is limited. In a pathology study (n=15), no incomplete infarct was found in WMH.^29^ Though many cross-sectional studies have found low cerebral blood flow (CBF) to be associated with higher WMH burden, the causality between low CBF and WMH is unclear.^35^ A longitudinal study (n=575) showed that more severe baseline WMH predated CBF decline over time rather than falling CBF predating WMH progression.^36^ In a postmortem study, some non-inflammatory, periventricular venulopathy was observed in periventricular WMH, suggesting that venous collagenosis might cause tissue damage by vasogenic oedema and impede ISF circulation.^31^ However, this theory remains to be confirmed in in vivo studies. Impaired BBB was noted in WMH areas in autopsies,^29^ ^30^ which was corroborated by studies using cerebrospinal fluid (CSF)/plasma albumin ratio^37^ and MRI.^38–41^ It is hypothesised that the disrupted BBB would result in leakage of fluid, plasma components and cells and eventually lead to perivascular inflammation, demyelination and gliosis. Indeed, the formation of WMH is likely to be multifactorial. Hypoperfusion, venous pathologies and BBB impairment might all play critical roles in WMH initiation or progression and interact with each other, but which one is the key initial factor remains unknown.

#### Lacunes

**Figure 3:**
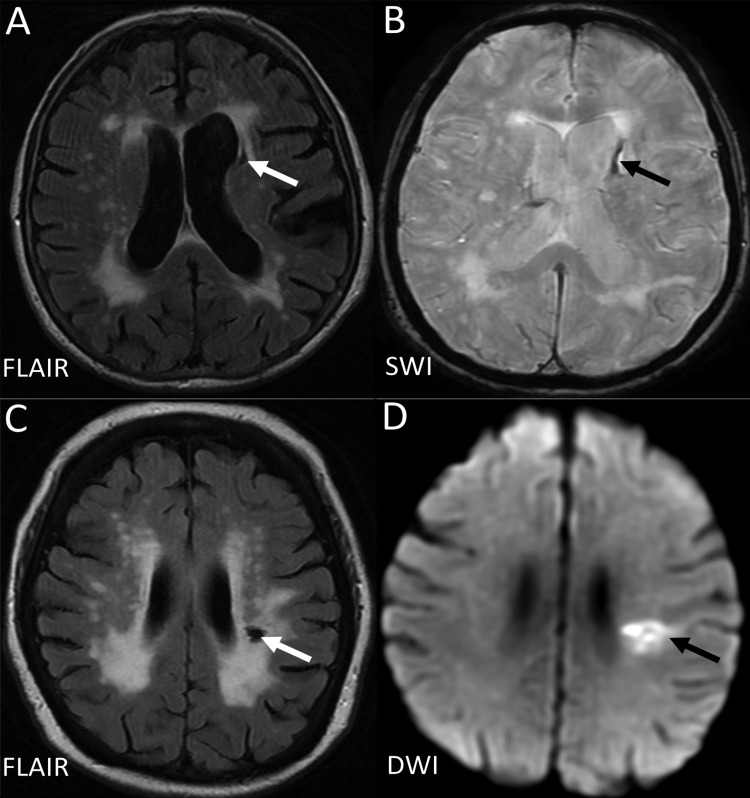
Example of MRIs of a lacune from a haemorrhagic source (A,B), and from a lacunar infarct (C, D). D (the DWI) is from the acute presentation (i.e. within a few days of the stroke), and C (the FlAIR) is weeks to months later when the lesion has cavitated. DWI, diffusion-weighted imaging; FLAIR, fluid-attenuated inversion recovery; SWI, susceptibility-weighted imaging.

The term ‘lacune’ was used by Fisher to describe a small fluid cavity in the brain which he thought was a healed lacunar infarct. Therefore, in CSVD research, it is very common that terms like ‘lacunar infarction’, ‘lacunar stroke’ and ‘silent brain infarct’ were used to refer to the CSF-filled cavities on brain MRI or autopsy.^42^ In fact, lacunes are not always ‘ischaemic’. They can also be the residual lesion of a small haemorrhage^43^ (figure 3). Also, it is common that many non-cavitated lacunar ischaemic strokes were not counted as ‘lacunar infarcts’. Therefore, in order to avoid more confusion, the term ‘lacune of presumed vascular origin’ was proposed to replace ‘lacune’ and the term ‘lacunar infarct’ should NOT be used to describe ‘lacunes’ any more.

Lacunes of presumed vascular origin are round or ovoid, subcortical, fluid-filled cavities with a diameter of 3–15 mm. These can occur without any prior symptoms, but can also result from a previous acute small subcortical infarct or haemorrhage^4^ (figure 1). PVS could also mimic lacunes when they are more than 3 mm in diameter.^44^ Large PVS might have also been miscounted as lacunes in many studies.^42^ Lacunes usually present as a hypointense ‘hole’ on FLAIR surrounded by a hyperintense rim which can help its differentiation from PVS. However, the rim can be absent in some cases and PVS within extensive WMH areas may appear as if surrounded by hyperintensities, so the insistence on a rim to differentiate lacunes from PVS is not helpful in practice. Nonetheless, it is important to distinguish between lacunes and PVS if possible, on size at least, because they represent different pathologies as well as differ in clinical associations and implications.

Although many lacunes might have lacked acute symptoms, when present in larger numbers they are associated with dementia, cognitive impairment, gait disturbance and increased risk of stroke.^5^ ^45^ ^46^ In the general elderly population, the prevalence of lacunes ranges from 8% to 28% (mean age=50–75 years).^5^ A systematic review suggests that silent brain infarcts (another term sometimes used for lacune) are more prevalent in the Asian than in the non-Asian population.^47^ However, it is noteworthy that most of these Asian studies were hospital-based, whereas all non-Asian studies were community-based; therefore, more relevant comparisons are needed to determine if the prevalence of lacunes and other CSVD features does differ between world regions and ethnic groups.

#### Perivascular spaces

PVS are the extension of subarachnoid spaces that surround cerebral microvessels.^48^ They are fluid-filled spaces that follow the course of a vessel through the brain parenchyma.^48^ PVS are usually microscopic and not detected on CT or conventional MRIs. When enlarged, PVS are commonly seen as hyperintense on T2 MRI, either punctuate with a diameter <3 mm if imaged perpendicular to the course of the vessel, or linear if imaged parallel to the course of the vessel^49^ (figure 1). PVS are most frequent in the inferior parts of the basal ganglia and centrum semiovale but can also occur in the brainstem. Though 3 mm has generally been considered as the cut-off diameter for distinguishing PVS from lacunes,^44^ occasional PVS could be larger and even cause a mass effect.^4^ PVS usually do not have a hyperintense rim on T2-weighted or FLAIR unless passing through a WMH area, which can help the discrimination between PVS and lacunes.

Whether PVS should be regarded as ‘lesions’ is still controversial, as their clinical significance remains unclear. Although a few PVS can be normal,^50^ numbers of PVS increased with advancing age and other features of CSVD.^51–54^ In some studies, more PVS were associated with increased risk of dementia or worse cognitive function or hypertension.^44^ ^55^ ^56^ The mechanisms underlying enlarged PVS are not well understood. In normal ageing and other neurological diseases like multiple sclerosis, PVS are associated with inflammatory markers.^57^ In CSVD, it might be a sign of impaired BBB.^39^ There is also a hypothesis that visible PVS are associated with a blockage of drainage of ISF,^58^ which might be attributed to increased vessel stiffness, as arterial pulsatility is thought to be a key driver of ISF drainage.^59^ They may also be a key conduit for drainage of brain interstitial metabolic products that occurs during sleep.^60^

#### Cerebral microbleeds

CMBs are regarded as small round and homogeneous foci of hypointensity on T2-weighted (gradient echo) MRI and susceptibility-weighted imaging (figure 1). In the very few studies of radiological–pathological correlation, perivascular hemosiderin-laden macrophages were found to underlie most of the CMBs shown on MRI. Other possible pathologies include old haematomas, intact erythrocytes and, very rarely, vascular pseudocalcification, microaneurysm and distended dissected vessels.^61^ Lipofibrohyalinosis and amyloid angiopathy are the most common vascular findings in relation to CMB. These two vasculopathies are thought to have different patterns of CMB distribution: CMBs in the basal ganglia, thalamus, brainstem and cerebellum are typically attributed to lipofibrohyalinosis, whereas amyloid angiopathy is more associated with lobar CMBs.^62^ However, some studies suggest that there may be more overlap and larger studies are awaited to confirm the specificity of CMB distribution for particular pathologies.

Most CMBs are asymptomatic; they can be found in healthy adults but are more often a marker of vascular risk factor exposure or amyloid deposition.^63^ In addition to its potential association with stroke, CMBs also contribute to cognitive impairment and dementia, and to transient neurological deficits.^64^ The prevalence of CMBs detected in community-dwelling participants in the Rotterdam Scan study (n=3979, mean age=60.3 years) and AGES-Reykjavik study (n=1962, mean age=76 years) was 11.1–15.3%^65^ ^66^ and increased with age.^66^ In patients with ischaemic stroke and non-traumatic intracerebral haemorrhage, the prevalence of CMBs could be as high as 33.5–67.5%.^63^ It seems that CMBs may be more common in the Asian than in the non-Asian population. However, the differences might be due to a higher proportion of hypertensive patients recruited in these Asian studies or more hospital-based than community studies.

It is unclear whether CMBs increase the risk of haemorrhage in patients receiving antiplatelet or anticoagulant or thrombolytic therapy and further discussion is outside the ischaemic focus of this review. We refer the reader to recent reviews on this topic^63^ ^67^ and note that randomised trials are needed to answer these questions.

#### Risk factors and causes of ‘silent’ CSVD

Increasing age is significantly associated with CSVD features; thus, age has to be controlled for while interpreting relevant studies. Modifiable risk factors including hypertension, hypercholesterolaemia, smoking and diabetes mellitus are also thought to be key risk factors in the pathogenesis of CSVD, particularly hypertension. However, the relationship between these risk factors and CSVD is complex. Lipohyalinosis, the typical vascular changes of CSVD, has long been thought to result from hypertension. The theory is supported by clinical evidence showing that hypertension is more prevalent in patients with WMH and that higher blood pressure was associated with more severe WMH.^68^ A recent study shows that vascular risk factors and large artery disease explained only 2% of the variance in WMH, leaving 98% of the variance unexplained, providing further evidence that WMHs are mostly non-atheromatous.^69^ This finding may give a clue as to why risk factor modifications so far have very limited effects on preventing WMH progression. Other important risk factors for CSVD include other high-risk lifestyles: lack of exercise, poor diet and smoking. High salt intake is associated with more severe WMH through causing high blood pressure, as well as by having direct effects on the endothelium.^70^ Current smoking is also an independent predictor of WMH progression^71^ and is associated with a high burden of combined CSVD features.^72^ Lack of exercise is a risk factor for having more WMH in later life, although it is not clear if active exercise programmes reduce WMH risk.^73^

### CSVD as a ‘whole-brain disease’

Common small vessel pathologies and BBB impairment were found in clinically evident and covert CSVD features, suggesting that CSVD should be regarded as a whole-brain disease rather than be treated separately as individual conditions. Small penetrating vessels and the endothelium, which forms the BBB, are diffuse in the brain. Various studies also demonstrate that all these CSVD features were associated with each other: patients with small vessel stroke (TOAST classification) or lacunar stroke (OCSP classification) had more WMH than those who had other stroke subtypes;^74^ ^75^ more than 90% of incident lacunes appeared at the edge of WMH or had a partial overlap with WMH in 365 patients with Cerebral Autosomal-Dominant Arteriopathy with Subcortical Infarcts and Leukoencephalopathy (CADASIL);^76^ visible PVS were frequently seen in patients with lacunar stroke, WMH and lacunes; CMBs were also associated with WMH and lacunar stroke.^63^ When counting the presence of any CSVD as the total CSVD score, patients with lacunar stroke had a significantly higher CSVD burden than those with cortical stroke.^72^

Why do some CSVD lesions cause stroke while others are ‘silent’? One explanation is the locations of lesions. A study using probability mapping shows that lesions presenting with stroke were predominantly located in or near the primary motor and sensory tracts, whereas silent lesions were mostly in the basal ganglia and centrum semiovale away from these main tracts.^77^ Another explanation could be the levels of vessels where the vascular pathologies happened. In general, disrupted BBB would enable plasma fluid components and blood cells to enter the vessel wall, leading to disintegration of the vessel wall and fibrin deposition. If this happens at arterioles where there is smooth muscle, the components deposited in the arteriolar wall could result in dilation and narrowing of the vessel lumen and vessel wall thickening, which would eventually precipitate inflammation, platelet adhesion, luminal occlusion and thus traditional infarct. However, at the capillary level where there is no smooth muscle between the epithelium and brain tissue, the leaky BBB would cause direct damage in the tissue, such as oedema and demyelination in white matter tracts. Further studies to assess changes over time in lesion development and symptoms are required to find out the reasons.

### CSVD as a ‘dynamic disease’

**Figure 4:**
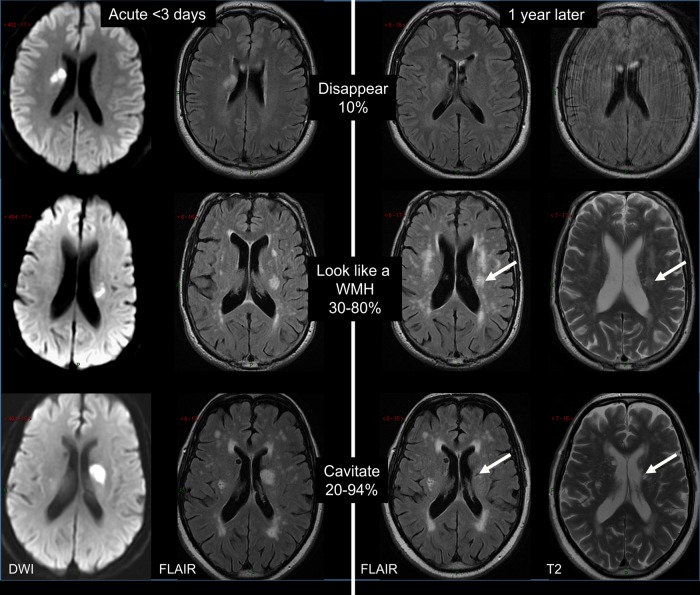
Long-term appearances of lacunar infarcts (arrows: old stroke lesion on the follow-up scans). DWI, diffusion-weighted imaging; FLAIR, fluid-attenuated inversion recovery; WMH, white matter hyperintensity.

There is increasing evidence showing that CSVD is more dynamic than originally thought. Lesions progress over time and the long-term outcome and impact on brain damage vary. Cavitation is not the only fate of acute lacunar ischaemic stroke.^78^ An acute lacunar ischaemic stroke can also disappear or resemble a WMH (figure 4). In a prospective study (n=90), definite cavitation (ie, that looked like a lacune) was only present in 20% of patients, and was marginally associated with increasing time from stroke onset to follow-up scans. A large proportion of lacunar lesions remained looking like WMH. Thus, only calculating cavitated lacunes could lead to a large underestimation of lacunar ischaemic stroke burden. Similarly, WMH burden is likely to be overestimated without previous scans of index stroke lesions.

The evolution of WMH also varies. The single strongest predictor of WMH progression is high baseline WMH,^79^ ^80^ with little progression in punctate WMH but rapid progression in confluent WMHs.^81^ The Austrian Stroke Prevention Study, a community-based study, reported WMH progression in about 18% of participants with vascular risk factors.^79^ WMH can also cavitate to take on the appearance of lacunes and they can also disappear—these dynamic features are only now being realised. Though early microstructural impairment could be detected in NAWM contouring WMH, not all NAWM will eventually develop into WMH.^82^ The level of NAWM deterioration was also strongly associated with WMH severity, regardless of distance from the WMH.^32^

The variance in long-term changes of CSVD lesions might reflect different pathologies underlying the similar appearance on imaging, for example, reversible lacunar ischaemic stroke lesions versus those that cavitated, or NAWM in patients with mild WMH versus in extensive WMH. Serial imaging studies using advanced techniques like cerebral vascular reactivity, BBB and CBF imaging and use of higher fields, for example, 7 tesla MRI might help differentiate these changes.^83^

### Treatments for CSVD

Management of traditional risk factors is still the main approach for treating or preventing CSVD, despite the fact that most of these treatments have not yet shown ideal effects on long-term outcome. Antihypertensive treatment produced contradictory results: it reduced WMH progression in some observational studies^84^ but showed little or no effects in randomised controlled trials.^85^ ^86^ Although hypertension has been reported to be highly associated with CSVD, other factors may be involved or be influenced by genetic factors,^87^ yet more evidences are required. Likewise, most lipid-lowering treatment had neutral results in preventing WMH, like pravastatin.^88^ Post hoc analysis of a 2-year follow-up study from Hong Kong showed that statins might be able to delay WMH progression in patients with severe baseline WMH.^89^ Statins might also have other therapeutic effects including anti-inflammatory and proendothelial activities.^90^ Likewise, subgroup analysis of the VITAmins TO Prevent Stroke (VITATOPS) MRI substudy shows that vitamin B supplementation may reduce WMH progression in patients with severe baseline CSVD.^91^

Studies of treatment specifically targeting lacunar stroke are limited.^90^ Apart from the SPS3 trial, there are very few clinical trials of antiplatelets where the results were reported by stroke subtype, and, except trials of cilostazol^92^ ^93^ which has weak antiplatelet effects,^94^ are especially scarce in Asian populations. Although some trials reported the proportion of lacunar stroke in their study population, the diagnostic criteria varied considerably and the results were not always reported by subgroup. A systematic review of randomised trials found that any single antiplatelet appeared beneficial for secondary prevention of lacunar stroke,^95^ but the SPS3 trial showed that long-term dual antiplatelet treatment doubled the risk of bleeding without reducing the risk of stroke recurrence in patients with recent lacunar stroke. Also, blood pressure lowering did not show significant reduction in recurrent lacunar stroke in the SPS3 trial, although it was consistent with a modest benefit.^96^

Prevention and treatment of CSVD in the future should consider targeting the BBB, brain endothelium and microvascular function. There are multiple potential endothelial targets, such as the nitric oxide/cyclic guanylate monophosphate (cGMP) system and prostacyclin/cyclic AMP (cAMP) system.^90^ Therefore, interventions that could induce cAMP or cGMP or reduce their degradation appear promising. There are several licensed drugs that have these properties like some nitric oxide donors and phosphodiesterases-5 inhibitors,^90^ while others are still in development. More experimental studies should be encouraged. However, in the meantime, management of these traditional risk factors according to guidelines should still be encouraged except to avoid long-term dual antiplatelet drugs.

In conclusion, CSVD is not just a collection of individual brain lesions, but is both a ‘dynamic’ and ‘whole-brain’ disease. All CSVD subtypes might share some common intrinsic CSVD aetiologies. Some pathological changes at the early stage of the disease could be reversible, but will gradually worsen and become irreversible as the damage in vessels and tissues accumulates. Modification of traditional risk factors and a healthy lifestyle are currently the most important prophylactic and therapeutic approaches for CSVD indefinitely and until more specific treatments are available. Apart from the trials of cilostazol which have mostly been conducted in China or Japan, in general, large clinical trials of CSVD treatments targeting the Asian population are lacking, especially in lacunar stroke. Community-based studies of CSVD prevalence and progression are also needed to determine if prevalence genuinely differs in different world regions or ethnic groups. Future studies in CSVDs should stratify by stroke subtype and by MRI diagnosis and measure risk factors carefully. Clinical trials and experimental studies targeting endothelium and BBB integrity should be pursued.

## Supplementary Material

Web abstract
