# Supplementary material for: Update on cerebral small vessel disease: a dynamic whole-brain disease
Source: Stroke Vasc Neurol. 2016 Oct 25;1(3):83–92. doi: 10.1136/svn-2016-000035 (PMC5435198; doi:10.1136/svn-2016-000035)
Supplement: Web abstract [file svn-2016-000035-s1.pdf]

Update on cerebral small vessel disease – a dynamic, whole-brain disease

Yulu Shi （施余露）；Joanna M Wardlaw

Affiliations and address:

Centre for Clinical Brain Sciences, University of Edinburgh, Edinburgh, United Kingdom (Y.S., J.M.W.); Department of Neurology, Zhongnan Hospital, Wuhan University, Wuhan, China (Y.S.).

Correspondence to: Prof Joanna M Wardlaw, Centre for Clinical Brain Sciences, University of Edinburgh, Edinburgh, EH16 4SB, United Kingdom. Joanna.Wardlaw@ed.ac.uk. Phone: +44-(0)131-465-9588

Title in Chinese: 小血管病研究新进展——一个动态的全脑性疾病

Abstract in Chinese

脑小血管病是老年人中最常见的神经系统疾病。它不仅导致脑卒中和痴呆，同时还与抑郁和步态问题密切相关。由于很难在体直接观察小血管病理改变，目前其诊断有赖于间接的影像学表现，包括脑白质高信号、腔隙性脑梗塞、陈旧腔隙、脑微出血、可见的血管周围间隙以及很多出血性卒中。然而，由于用来形容这些病变的术语和定义众多且杂，为解读既往的小血管病研究造成诸多困扰，因此我们提倡在未来研究中统一使用标准化的术语。一直以来，这些小血管病变都被看作不同类型的病灶，但越来越多的研究显示它们也许共有一些微小血管内固有的病理改变；同时，因为微小血管散在于全脑，小血管病应被视为一种“全脑”疾病。目前由于对小血管病机理的认识尚存局限，单种抗血小板聚集药物（针对腔隙性脑梗塞）和控制传统血管危险因素依旧是小血管病最重要的治疗和预防措施。但是，越来越多的证据提示我们未来防治研究可以着眼于保护内皮和血脑屏障。亚洲人群的小血管病流行病学表现也许异于西方人群（目前小血管病和卒中的诊疗指南主要来源于后者），但仍需要更多基于社区人群的数据以及在未来研究中对卒中明确分型才能得到更清晰的答案。
